# Identification of Exploration and Exploitation Balance in the Silkmoth Olfactory Search Behavior by Information-Theoretic Modeling

**DOI:** 10.3389/fncom.2021.629380

**Published:** 2021-02-01

**Authors:** Cesar A. Hernandez-Reyes, Shumpei Fukushima, Shunsuke Shigaki, Daisuke Kurabayashi, Takeshi Sakurai, Ryohei Kanzaki, Hideki Sezutsu

**Affiliations:** ^1^Department of Systems and Control Engineering, Tokyo Institute of Technology, Tokyo, Japan; ^2^MHPS Ltd., Takasago, Japan; ^3^Department of Systems Innovation, Osaka University, Osaka, Japan; ^4^Department of Agricultural Innovation for Sustainability, Tokyo University of Agriculture, Atsugi, Japan; ^5^Research Center for Advanced Science and Technology, The University of Tokyo, Tokyo, Japan; ^6^Transgenic Silkworm Research Unit, National Agriculture and Food Research Organization, Tsukuba, Japan

**Keywords:** *Bombyx mori*, infotaxis, olfaction, ethology, adaptive-behavior, exploration-exploitation

## Abstract

Insects search for and find odor sources as their basic behaviors, such as when looking for food or a mate. This has motivated research to describe how they achieve such behavior under turbulent odor plumes with a small number of neurons. Among different insects, the silk moth has been studied owing to its clear motor response to olfactory input. In past studies, the “programmed behavior” of the silk moth has been modeled as the average duration of a sequence of maneuvers based on the duration of periods without odor hits. However, this model does not fully represent the fine variations in their behavior. In this study, we used silk moth olfactory search trajectories from an experimental virtual reality device. We achieved an accurate input by using optogenetic silk moths that react to blue light. We then modeled such trajectories as a probabilistic learning agent with a belief of possible source locations. We found that maneuvers mismatching the programmed behavior are related to larger entropy decrease, that is, they are more likely to increase the certainty of the belief. This implies that silkmoths include some stochasticity in their search policy to balance the exploration and exploitation of olfactory information by matching or mismatching the programmed behavior model. We believe that this information-theoretic representation of insect behavior is important for the future implementation of olfactory searches in artificial agents such as robots.

## 1. Introduction

Odor source localization is a search problem that requires fast decision-making based on sporadic and stochastic detection of chemical particles. Despite the challenge of turbulent and dilute plumes that often have a complex spatio-temporal structure (Mafra-Neto and Cardé, 1994; Celani et al., 2014), insects such as the fruit fly (van Breugel and Dickinson, 2014) and various species of moths (Vickers, 2005) rely on olfactory searches to conduct essential behaviors such as searching for food or potential mates. The high performance that insects show on such a complex search problem despite their simple brain motivates researchers to further analyze and understand the decision processes that these insects execute when conducting olfactory searches (Baker et al., 2018).

With this motivation, our research group has analyzed the olfactory behavior of the male silk moth *Bombyx mori* (lepidoptera: bombycidae). Despite having wings, this insect is unable to fly, and has a body that is on average 30 mm long and 10 mm wide. It has two antennae of approximately 6 mm in length on its head. This insect has been widely employed to analyze olfactory behavior because it exhibits only one action: It walks only when it detects a pheromone (*Bombykol*) released by its female counterpart (Obara, 1979). Such behavior consists of a series of maneuvers called a “surge,” “zigzag,” and “loop.” This sequence of maneuvers has been approximated to a mean-response model denoted as “programmed behavior” (Kanzaki et al., 1992).

**Figure 1:**
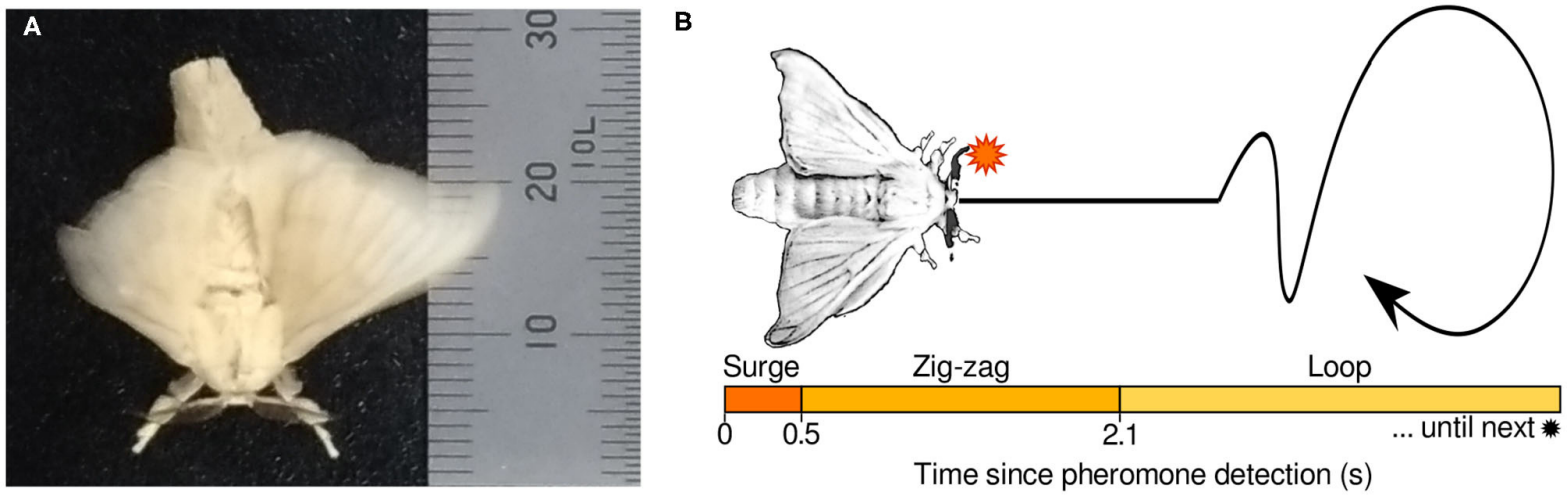
**(A)** Specimen of a male silk moth pictured next to a ruler in mm. **(B)** Conceptual diagram of the “programmed behavior” model of the male silk moth behavior.

Based on the mean durations of the surge, zigzag, and loop maneuvers, the programmed behavior has been algorithmically defined as follows: first, immediately after a pheromone stimulus, the moth advances in a straightforward manner through a *surge* motion. Then, if there is an absence of pheromone detections, the moth moves on a *zig-zag* pattern, trying to detect pheromones again. Finally, if the pheromone remains undetected, the moth transitions into a *loop* motion until the next detection. A diagram of the programmed behavior is shown in Figure 1. Because the silk moth is motionless by default and only elicits its programmed behavior after the first pheromone hit, this search strategy has been labeled as “reactive” by Voges et al. (2014). Despite the simplicity of this sequential pattern, the male silk moth can effectively locate females with remarkable efficiency.

However, this model does not reflect how the motions of the moth vary in response to fine spatio-temporal fluctuations of the odor plume and individual differences among specimens. In previous studies, such variability was investigated by identifying maneuver transitions with machine learning (Shigaki et al., 2018b) and fuzzy logic (Shigaki et al., 2019b). Although these studies succeeded in identifying deviations from the programmed behavior, they relied on data from electro-physiological signals obtained from implanting electrodes in the wing muscles or brain of the silk moth; however, electrode implantation is technically challenging and risks degrading the tissues of the moth. Therefore, an analysis method that allows modeling adaptive olfactory behavior from non-intrusive experimental measurements is necessary.

To identify adaptive olfactory behavior, recent studies have used the information-theoretic framework of infotaxis, which was first proposed by Vergassola et al. (2007). A recent study by Pang et al. (2018) investigated the features of odor encounters that modulate the intensity of upwind turns in the fruit fly *Drosophila melanogaster* and the mosquito *Aedes aegypti*. The authors found through simulations that, compared to a *centerline inferring* odor source search algorithm, infotaxis produced trajectories that were more similar to those of the actual animals, in the sense that they exhibit weaker upwind turns later in a sequence of odor encounters. Similarly, Calhoun et al. (2014) recently demonstrated the possibility of using infotaxis to model the multi-stage foraging behavior of the nematode *Caenorhabditis elegans*. In their paper, the authors showed that infotaxis-like search strategies, which minimize the entropy of the probability distribution of odor source locations, reflects both the “local” and “global” stages of the *C. elegans* foraging behavior.

In this paper, we investigate the potential causes of variability in the behavioral maneuvers of the silk moth *B. mori* by using a non-invasive experimental method and an infotaxis-based model similar to those described in recent studies. We measured the silk moth trajectories and input stimuli data with a tether, a two-dimensional treadmill, and a virtual odor plume. To ensure accurate and reproducible stimuli, we used optogenetic silk moths that react to the impulses of blue light in the same way as with pheromone particles. We modeled the trajectories and stimuli measurements as infotaxis agents and found that; maneuvers that mismatch the *programmed behavior* model correspond to higher expected information rewards regarding the location of the source. In summary, we believe that this paper demonstrates the possibility of using non-invasive experimental measurements and infotaxis-based modeling to identify variability in the olfactory behaviors of the male silk moths.

This paper is structured as follows: section 2 states the research questions of this paper. Section 3 describes the usage of optogenetic silk moths, the experimental virtual reality system to measure their behavior, and how to model it as infotaxis agents. Section 4 shows the results of the behavior measurement experiments and calculations of the information entropy of infotaxis-modeled silk moths. Section 5 discusses the contributions of this study and possible future areas of research.

## 2. Problem Statement

In this paper, we look for possible causes of adaptive mechanisms in the olfactory behavior of the silk moth, which are not represented in the programmed behavior model. Specifically, we investigate the following two hypotheses:

- Are deviations from the programmed behavior motivated by higher information gains?
- Can a probabilistic framework such as infotaxis explain how the male silk moth balances exploration and exploitation of olfactory information?

To test the first hypothesis, we need to measure the behavior of the silk moth in an olfactory environment that can be accurately reproduced in each experimental run. Therefore, in this paper we utilize a “virtual reality” behavioral measurement system in which we can subject moths to virtual odor plumes and measure their motor response to odor stimuli. However, such a system faces the challenge of an accurate stimulation of the moth antennae. In other words, stimulating the antennae with gaseous pheromone particles results in uncertain stimulation because such particles diffuse in the air; hence, they do not produce stimuli with the same intensity or duration each time. To overcome this, we employed genetically modified silkmoths that elicit their normal olfactory behavior response when subjected to a blue light stimulus at their antennae; thus, we can present reproducible olfactory inputs.

To test the second hypothesis we modeled the trajectories of silkmoths as an agent that minimizes the information entropy of its probabilistic belief of the location of an odor source. Such a maximally informative agent is based in the infotaxis algorithm (Vergassola et al., 2007). We related the decrease in entropy of the infotaxis-modeled moth to the time steps in which the moth behavior matched or mismatched the programmed behavior model. Finally we determined whether infotaxis can explain the exploration-exploitation strategy of the silk moth behavior by evaluating the distribution of entropy reductions by either matching or mismatching behaviors.

## 3. Materials and Methods

Here, we describe our methodology for conducting olfactory search experiments with optogenetic male silk moths and a non-invasive behavior measurement system. We also describe the method we used to represent the silk moth trajectories as those of an infotaxis agent. The silk moth experiments in this study were examined and approved by the Tokyo Institute of Technology Gene Recombination Experiments Safety Management Committee.

### 3.1. Virtual Reality System for Measurement of Moth Behavior

**Figure 2:**
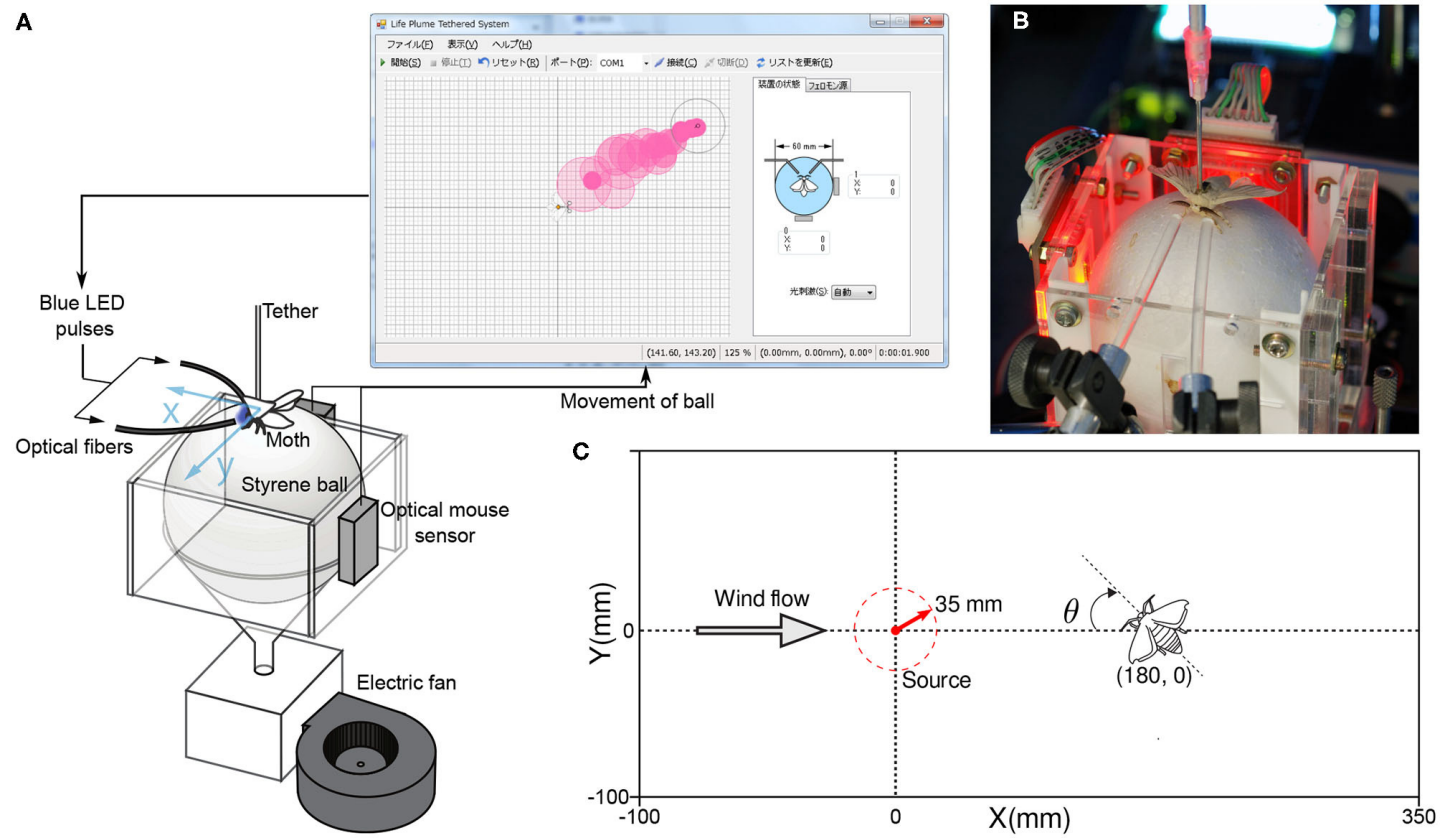
**(A)** A diagram of the behavioral measurement system used in our experiments. **(B)** An actual ChR2 moth used in the measurement system with optic fibers pointed at its antennae to present blue light stimuli. **(C)** The dimensions of the virtual environment to which we subjected the moths and their initial position.

We conducted non-intrusive behavioral measurements on tethered male silk moths. Although similar systems to measure the olfactory behavior of insects have been used in the past (Shigaki et al., 2018a, 2019b), in this study we ensure that odor stimuli are accurately presented by using optogenetic silk moths. Using genetically modified specimens that react to blue light stimuli in the same way as normal specimens react to the pheromone bombykol, allowed us to present stimuli accurately and with reproducibility. This is because gaseous pheromones diffuse in the air; therefore, not all stimuli present the same amount of pheromone molecules to the antennae of the moth. Furthermore, in this case, the response of the antennae is measured using an electroantennogram (EAG), which is technically challenging and subjected to electrical noise; in addition, damage to the antennae may occur. Our non-invasive behavior measurement system for the silk moth is shown in Figure 2, and fulfills the following purposes:

- Measuring the pose (*x*, *y*, θ) of the moths.
- Accurately presenting light stimuli to the antennae of the moth.
- Subjecting moths to a virtual odor plume to which we can alter the emission rate, wind speed, and other parameters.

To measure the pose of the silk moth, we fixed its back to a thin aluminum rod (Ø 2 mm; length 150 mm) with glue (G17 Bond, Konishi K.K., Osaka, Japan) and placed it on a polysterene sphere (Ø 60 mm), which served as a two-dimensional treadmill. When the moth walked, the sphere moved in response because it was being levitated by the flow of wind from a small fan (FW1251-1051C2ALARX, ARX, Wanchai, Hong Kong). The movements of the sphere were detected using two optical sensors, such as those found in a computer mouse (ADNS-5030, Avago Technologies, California, USA), at a sampling rate of 20 Hz. They were then translated into translational and rotational movements of the moth, that is, the pose.

We developed a virtual representation of an odor plume by modeling the dispersion of white smoke in a wind tunnel. First, we recorded videos of the dispersion of smoke. We also calculated the statistics of the position and intensity of the pixels in the smoke video. Based on these statistics, we programmed a random process that generates virtual circular puffs that match the intensity and transit the positions of the real smoke puffs in the video. An example of a virtual plume is shown in Figure 2A. In addition to the virtual representation of the odor plume, we also programmed a virtual representation of a silk moth. As in the real world, the virtual moth reacts to the virtual plume and travels toward its source. By using a virtual odor plume environment, we can tune parameters such as wind speed, emission rate, and particle lifetime. Tuning such parameters is particularly useful in infotaxis-based behavior modeling because it allows for faster testing of various plume structures and higher reproducibility; compared with real plume experiments. In summary, the following process describes the operation of our experimental device:

1. The moth in the virtual world encounters a puff of pheromone.
2. Blue light is shown to the real moth depending on which antenna of the virtual moth reacted.
3. The real moth moves after receiving the stimulus.
4. The movement of the real moth is sent to the virtual world.
5. The virtual moth reflects the movement of the real moth.
6. The loop is repeated until either the moth reaches the virtual source or until a predetermined time limit is passed.

### 3.2. Use of Optogenetic Moths for Accurate Antennae Stimulation

The presentation of accurate stimuli is important for the applied infotaxis-based analysis because updating the probability distribution of the source position; as well as the calculation of the expected entropy decrease, are directly affected by whether the agent experiences a hit or not at a given time step. In addition, reproducible odor stimuli are an overall useful property for an olfactory behavior measurement system because their duration and frequency can be finely tuned. Both properties have been reported to directly influence the olfactory behavior of moths (Celani et al., 2014) and other animals (Ache et al., 2016). To present olfactory stimuli to the moth, previous studies have presented pheromones from glass tubes placed directly in front of the antennae of the moth. However, the amount of pheromone particles that effectively reach the antenna varies owing to their gaseous nature.

To ensure that each stimulus has the same intensity and is accurately sensed by the antennae, we utilized genetically modified moths. These BmOR1-GAL4/UAS-ChR2 silk moths (ChR2 hereinafter); express channelrhodopsin-2 in their olfactory receptor neurons. As a result, they execute their olfactory search behavior when their antennae encounter blue light, rather than pheromone particles. This property has been used in previous studies to ensures that all stimuli are reproducible with the same intensity and duration (Shigaki et al., 2018b, 2019a). To activate channelrhodopsin-2, i.e., blue light sensitivity in these moths, we injected all-trans retinal (ATR) into their abdomen on the day before the experiments; because insects do not intrinsically possess ATR. All behavior measurement experiments were conducted from 9:00 to 17:00 to reduce circadian effects (Tomioka et al., 1993). It is reported that brain serotonin level increases in the daytime and that serotonin enhances pheromones sensitivities in the silk moth (Gatellier et al., 2004).

We generated stimuli for the ChR2 silk moths with LEDs (LBW5AP-JYKY-35-Z; Osram Opto Semiconductors), which produced blue light with a 470 nm wavelength and a light intensity of more than 1.6 mW/mm^2^. Such values of wavelength and light intensity have been reported to reliably produce olfactory search responses in ChR2 moths (Tabuchi et al., 2013). On each LED, we attached optical fibers of 3 mm in diameter to ensure that blue light was directed only to each antenna, as seen in Figure 2B. In addition, moths are unable to make yaw turns because their back is glued to an aluminum rod. The only rotation they are able to make is on their neck (see Supplementary Video). However, this neck rotation is very small and it does not decrease the sensibility or the amount of stimulation to the antennae.

### 3.3. Modeling the Silk Moth as an Infotaxis Agent

Infotaxis was first proposed by Vergassola et al. (2007) as an odor source search algorithm for turbulent environments. In this algorithm, a point-mass agent is located at a position **r** and searches for an odor source by iteratively reducing its uncertainty about the distribution of possible source locations **r**_*src*_. The agent has knowledge of its trajectory, $T_{t}$, which contains its sequence of positions as well as the odor “hits” it has experienced throughout the search. The agent also maintains a probability map *P*(**r**_*src*_|$T_{t}$) or “belief” (Thrun et al., 2005) about the location of the source. This belief spans all possible locations of the source **r**_*src*_ that in both the original infotaxis study and the present paper, consist of a two-dimensional lattice of discrete locations. The certainty of the belief *P*(**r**_*src*_|$T_{t}$) is represented by Shannon's entropy as in Equation (1):

(1) $$\begin{array}{l} S_{t}=S\left[P\left({\text{r}}_{src}|T_{t}\right)\right]=-\underset{{\text{r}}_{src}}{\sum }P\left({\text{r}}_{src}|T_{t}\right)\ln\left(P\left({\text{r}}_{src}|T_{t}\right)\right) \end{array}$$

The goal of infotaxis is to minimize the entropy of the belief *P*(**r**_*src*_|$T_{t}$); therefore, at every time step, the agent calculates the expected change of entropy by moving from its current position **r**_*t*_ to a future position **r**′ as defined in Equation (2).

(2) $$\begin{array}{l} E\left[\Delta S\left({\text{r}}_{t}\mapsto {\text{r}}^{\prime }\right)\right]=p^{*}\Delta S^{*}+\left(1-p^{*}\right)\Delta S \end{array}$$

**Figure 3:**
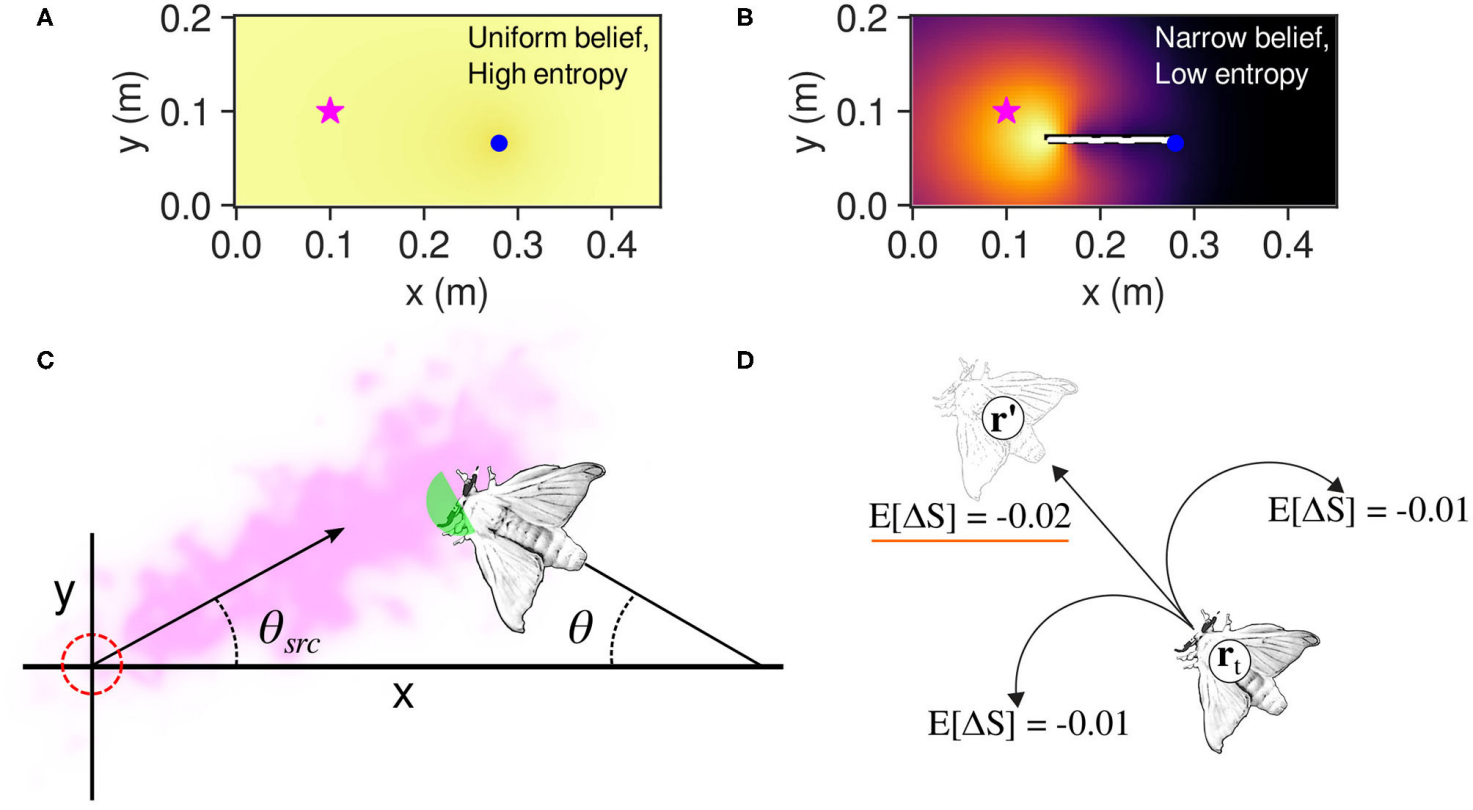
**(A)** An agent (blue dot) at the start of an infotaxis search. Each cell of the map has the same probability of being the odor source; thus, the entropy is maximal. **(B)** An agent that has narrowed down the probability distribution of the source location to an area near the actual source (star symbol). In this case, the information entropy of the belief is low. **(C)** How a silkmoth is modeled as a point-mass agent for infotaxis calculations. In this illustrative example, only the green area will react to pheromone particles owing to the “wingflap effect” i.e., when cos(π − θ + θ_*src*_) > 0. **(D)** The adaptation of the infotaxis navigation policy to a silkmoth. In this case, moving forward from position **r**_*t*_ to **r**′ yields more expected entropy decrease than rotating. Please note that a more negative value is more desirable because it would narrow down the possible locations where the odor source is located.

Where p^*^ is the probability of finding the source at **r**′, and ΔS^*^ and Δ*S* are the change in entropy if the source is found or not found at **r**′, respectively. The agent then executes the move ${\text{r}}_{t}\mapsto {\text{r}}^{\prime }$ with the largest negative value of *E*[Δ*S*], or; in other words, the move that causes the greatest reduction of uncertainty in the agent's probability map of the possible source locations. Figures 3A,B show conceptual representations of the agent's belief as well as the effect of odor detections on such belief. Detailed derivations of the infotaxis formulae are presented in Appendix A of this paper.

We modeled the body of the silk moth as a point agent with a radius of 10 mm (half of its average body length). We reduced the three degrees of freedom of the moth to (*x*, *y*) coordinates because an infotaxis agent moves in a two-dimensional grid ignoring the orientation. Furthermore, we considered as odor hits only those that occurred when the moth was facing upwind, that is, when cos(π − θ + θ_*src*_) > 0 (see Figure 3C), where θ and θ_*src*_ are the angle of the moth and the plume's centerline, respectively. We considered this capture region because real moths limit the odor hits to those coming from the front by flapping their wings (Loudon and Koehl, 2000).

### 3.4. Classification of Variability in the Moth Behavior

**Figure 4:**
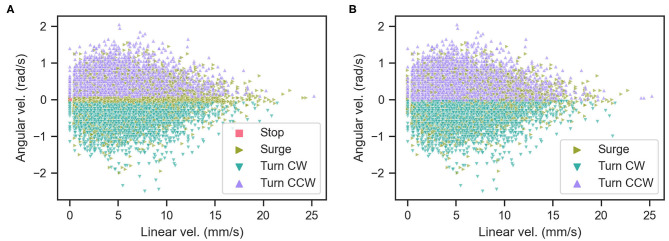
Classification of moth actions by **(A)** the kinematic criteria and **(B)** the temporal criteria.

We determined whether the behavior of the silk moth matches the definition of the programmed behavior (Kanzaki et al., 1992) by comparing it to the definition of Minegishi et al. (2012). Accordingly, we classified the maneuvers of the silk moth by simply considering the time elapsed since the last odor hit, which we call “blank duration” τ_*b*_ as in Celani et al. (2014). We also classified maneuvers according to both τ_*b*_ and the moth's linear and angular velocities (*v* and ω, respectively) based on Minegishi et al. (2012). We denote the first and second classification as “temporal” and “kinematic,” respectively. Table 1 shows a comparison of both schemes used to classify maneuvers and Figure 4 shows the result of using each scheme. The blank duration threshold of 500 ms in the “temporal” classification of Table 1 was selected because this is the average duration of surge motions after an odor hit as reported in Kanzaki et al. (1992). Throughout all olfactory search experiments, we classified the moth maneuvers by both schemes and labeled the state of the moth at each time step as “matching” if it matches the criteria of both schemes and “mismatching” if it only matches the “kinematic” criteria.

**Table 1:** Definitions for the maneuvers of silk moths when classified by either a temporal or a kinematic state.

	**Temporal**	**Kinematic**
Surge	τ_*b*_ ≤ 500 ms	τ_*b*_ ≤ 500 ms and *v*>0 or
		τ_*b*_ > 200 ms and |ω| <5deg/s
Rotate	τ_*b*_ > 500 ms	τ_*b*_ > 500 ms and |ω|> 0
Stop	Otherwise	Otherwise

To determine whether “mismatching” behaviors are motivated by higher information gains, we analyze the value of the entropy change Δ*S* and the expected entropy change *E*[Δ*S*] regarding the rate of odor hits and the cumulative odor hits experienced by moths over a search. We are particularly interested in these variables because recent studies identified that they influence the decision-process of olfactory behaviors (Celani et al., 2014; Pang et al., 2018). We also evaluate whether the distribution of Δ*S* is different for “matching” and “mismatching” behaviors with a two-sample Kolmogorov-Smirnov test and by comparing their histograms. In addition, we calculate the cumulative density function (CDF) of Δ*S* and *E*[Δ*S*] to specifically determine whether “mismatching” behaviors have a higher probability of obtaining larger negative values of those variables, that is, greater information gains. Finally, we calculate the root mean squared error (RMSE) between the values of Δ*S* and *E*[Δ*S*] to determine what type of behavior is more similar to infotaxis, regarding the rate of odor hits and the cumulative sum of hits, which are our variables of interest. The following section presents the results of the calculations of Δ*S* and *E*[Δ*S*] regarding hit rate and cumulative hits, the histograms and CDFs, and the RMSE of “matching” and “mismatching” behaviors.

## 4. Results

Here, we present the results of the VR odor source search experiments using optogenetic silkmoths. First, we present the trajectories of the moths as well as their information entropy. We then show the statistics of the matching versus mismatching states, followed by the relationship between those two states and the expected decrease in information entropy for each.

### 4.1. VR Olfactory Search Experiments

**Figure 5:**
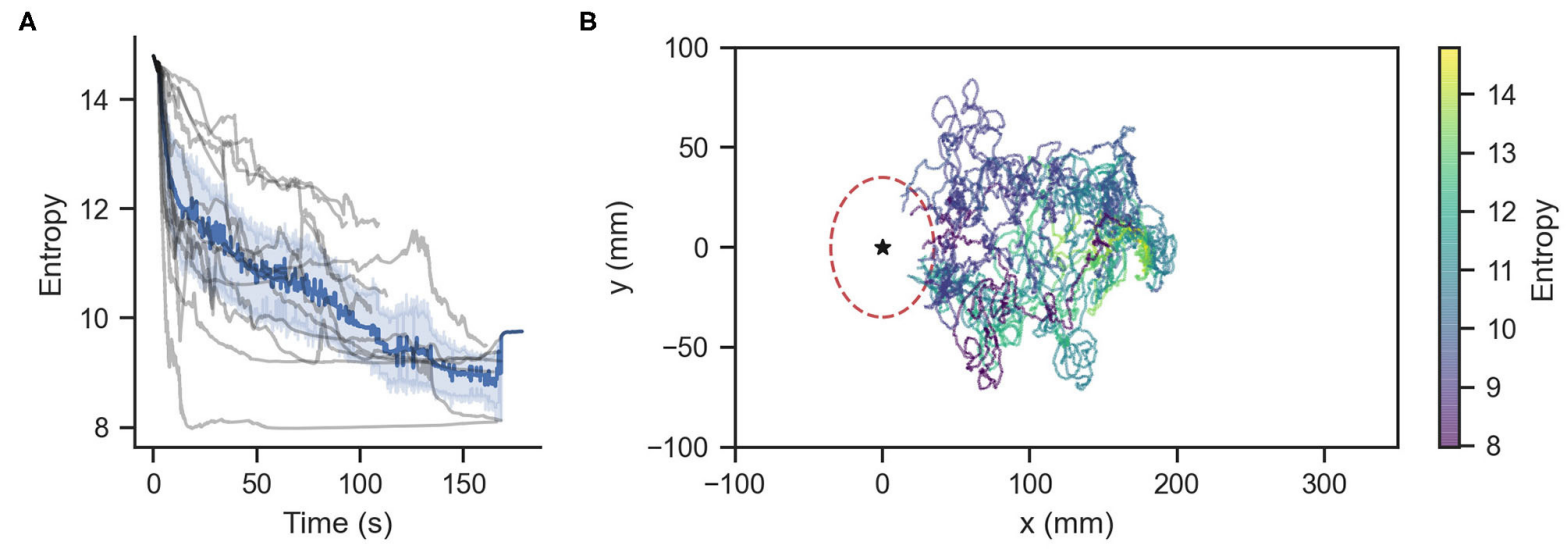
**(A)** Information entropy of infotaxis-modeled silkmoths. Gray lines represent each of the 12 runs that found the odor source. The blue line represents the average entropy. **(B)** Trajectories of the successful experimental runs. The star symbol represents the pheromone source.

We subjected ChR2 silkmoths to olfactory search experiments. We conducted 20 trials in which the moth searched for a pheromone source in a 350 mm long by 200 mm wide virtual environment where the wind was blowing in the positive *x*-direction at a mean speed of 0.1 m/s. The initial position of the moth in the virtual environment was (*x*, *y*, θ) = (180, 0, −π/6), where θ is in radians. Moths searched for a source located at (*x*, *y*) = (0, 0) by entering a radius of 35 mm around it under a time limit of 180 s. The mean ± std. dev. of the time required to reach the source was 73.92 ± 46.5 s. Figure 5A shows the information entropy for the experiments where moths found the pheromone source. The solid line represents the average value, the shaded range represents the standard deviation, and the gray lines show the value for each trial. Figure 5B shows the moth trajectories of these successful trials. The color gradient represents the value of the information entropy. Table 2 shows the statistics of the matching and mismatching moth states. Surge (temporal) and Rotate (kinematic) represent the proportion of time taken when the silk moths exhibited a mismatching state over the entire duration of the search experiments. In total we conducted 20 experiments with 10 specimens. Out of these, 12 trials from six specimens successfully found the odor source under the time limit; thus achieving a success rate of 60.0%. We considered only the data from the successful trials for the classification of matching and mismatching behaviors.

**Table 2:** Normalized counts of each maneuver taken by the moths.

	**Temporal**	
**Kinematic**	**Surge**	**Rotate**
Surge	0.1597 ± 0.07	0
Rotate	^**^0.1939 ± 0.11	0.6464 ± 0.19

*The “kinematic” classification scheme is based on the linear and angular velocities of the moths. The “temporal” scheme is based on the time since the last odor hit. The values with the asterisks indicate “mismatching” behaviors*.

### 4.2. Relationship Between Behavior Variability and Information Gains

**Figure 6:**
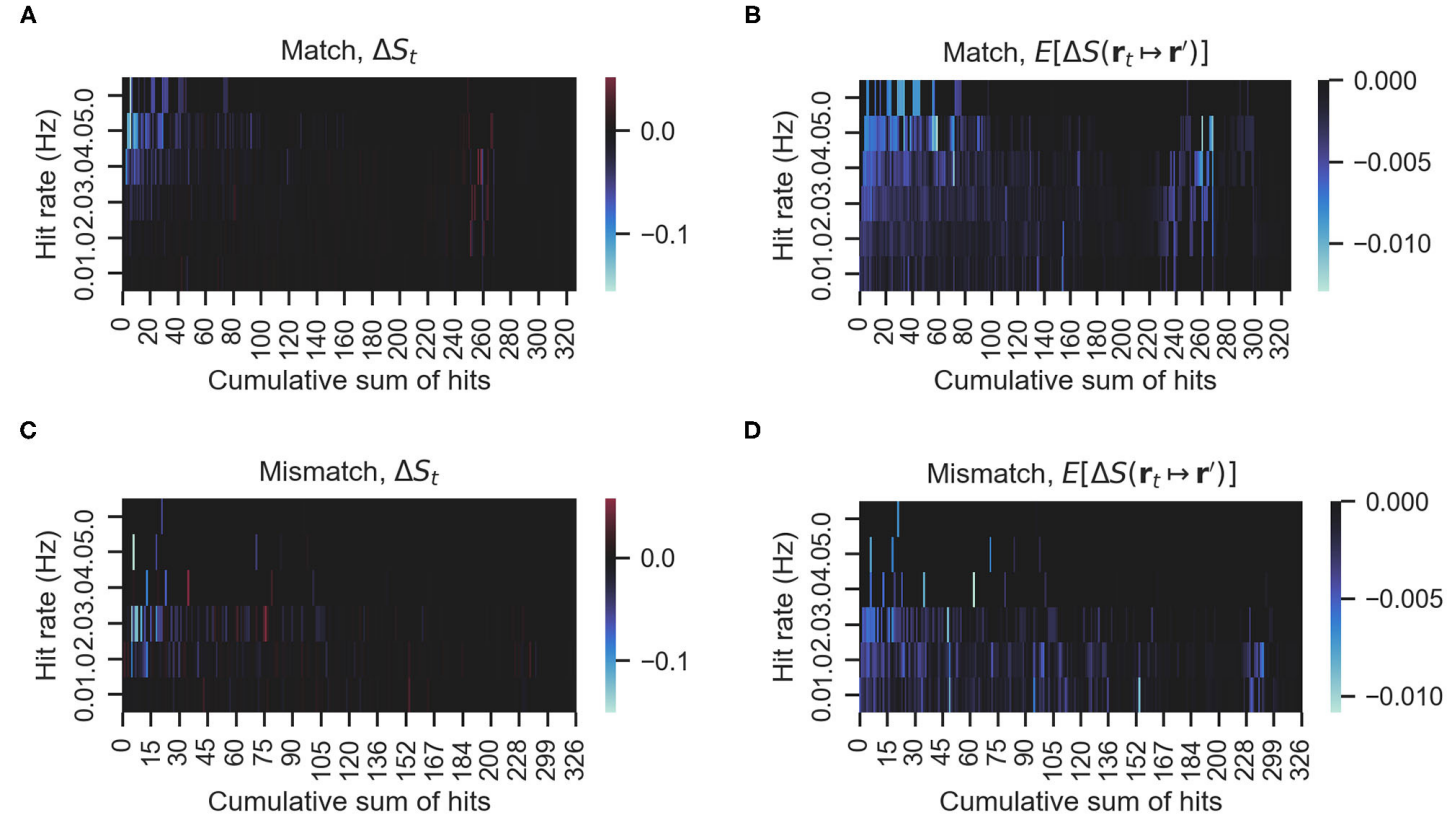
**(A,C)** The actual rewards obtained by either matching or mismatching behavior. **(B,D)** The expected rewards. Blue hue indicates more entropy decrease, that is, greater information rewards. Red hue indicates the opposite. In this figure, Δ*S*_*t*_ indicates the actual entropy change, in other words, *S*(**r**_*t*+1_) − *S*(**r**_*t*_). *E*[Δ*S*] indicates the expected entropy change for all possible actions (i.e., moving from **r**_**t**_ to **r**′).

**Figure 7:**
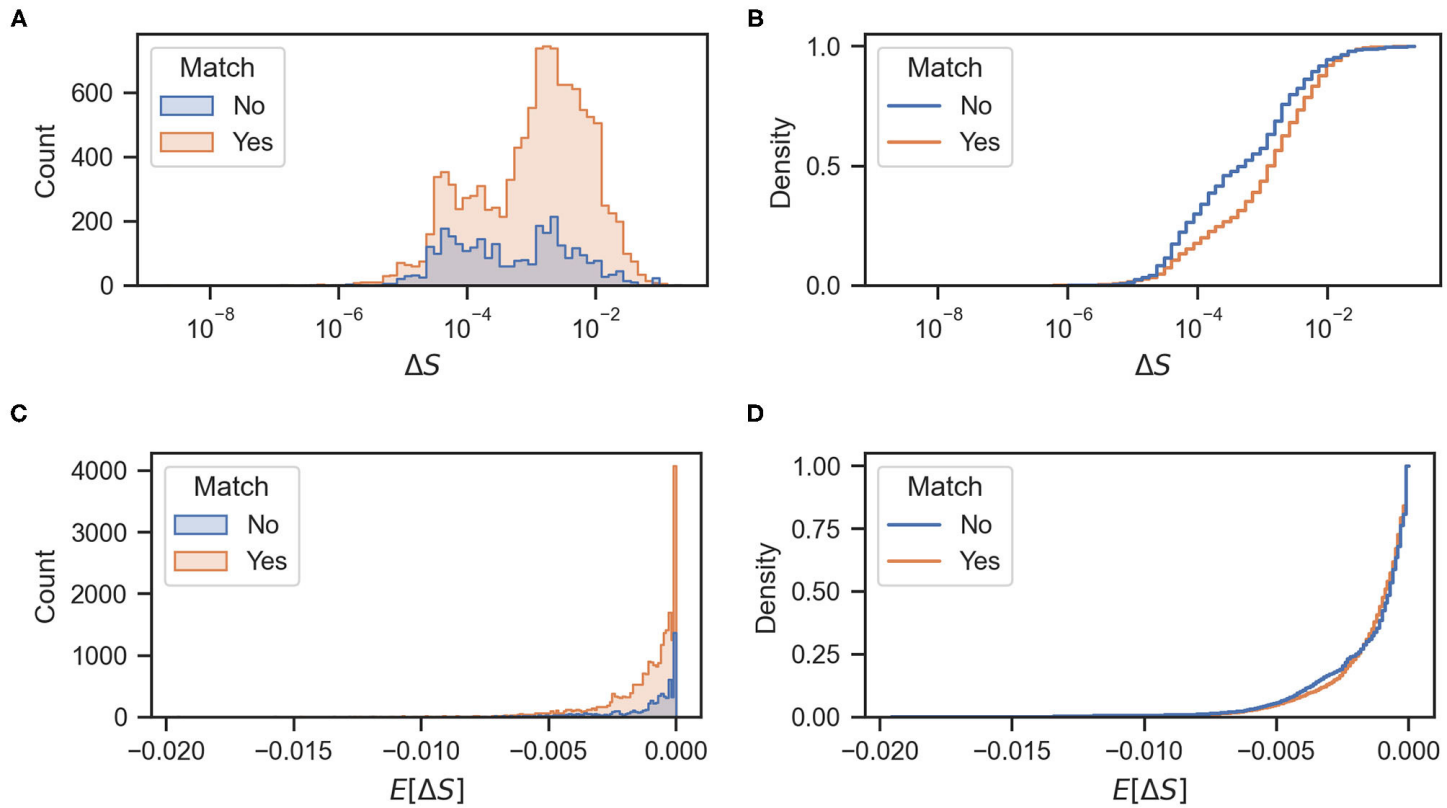
**(A,C)** Histograms of actual and expected rewards, respectively. **(B,D)** Cumulative density functions of the actual and expected rewards, respectively.

We investigated whether there is a relationship between mismatching maneuvers and a higher expected decrease in entropy $E\left[\Delta S\left({\text{r}}_{t}\mapsto {\text{r}}^{\prime }\right)\right]$. Figure 6 shows the actual rewards Δ*S* and expected rewards *E*[Δ*S*] of the match and mismatch behaviors. As can be seen in Figures 6A,C, matching and mismatching behaviors generate large decreases in entropy at low or high hit rates, respectively. In addition, the matching behaviors generated penalties (entropy increase) at high numbers of accumulated hits. Please note that entropy is non-monotonous (Hajieghrary et al., 2016; Rodríguez et al., 2017) and can increase on detection to non-detection sequences since the agent's belief is narrowed by the detection but broadens again at the non-detection. Figures 6B,D show that the expected rewards are greater at low or high hit rates for mismatching and matching behaviors, respectively. Figures 7A,C show histograms of the actual and expected rewards, respectively. We validated the statistical difference in the distributions of the matching and mismatching states (Kolmogorov-Smirnov test *p* < 0.01).

**Figure 8:**
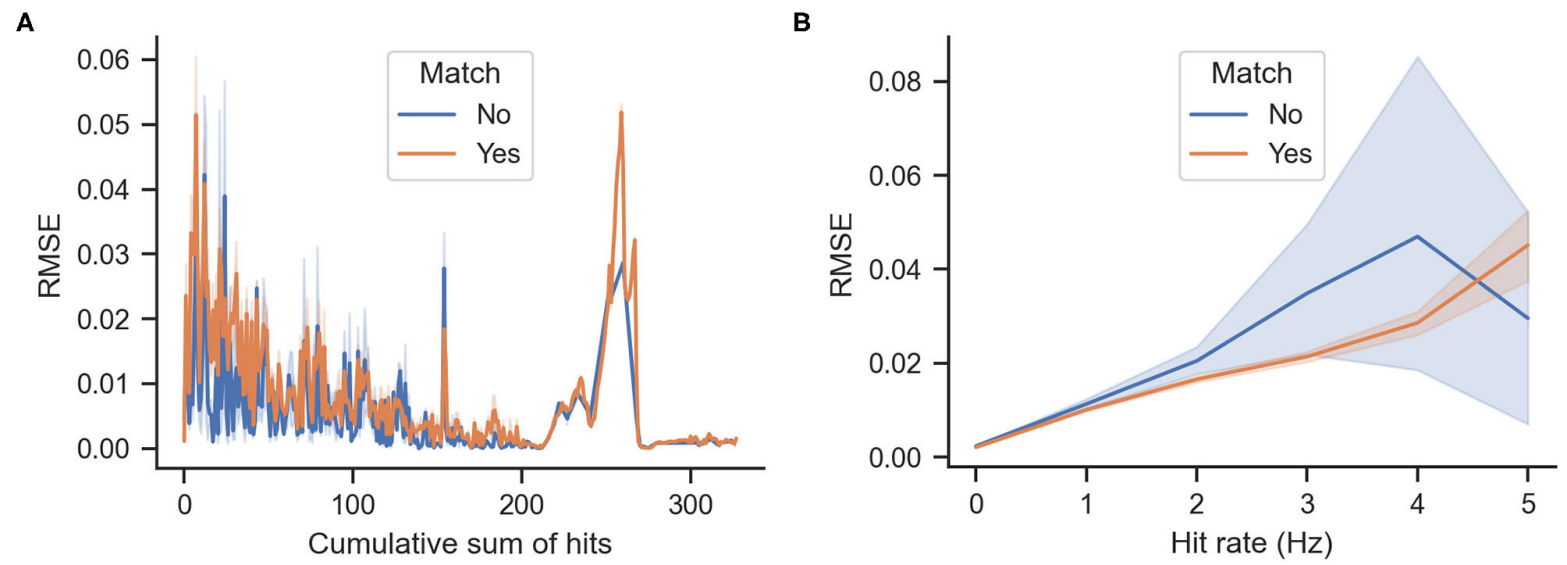
Root mean squared error (RMSE) between actual and expected rewards. Lower values indicate that the expected reward calculated by Equation (2) matches the actual rewards Δ*S*. **(A)** RMSE against the accumulated odor hits of the agent over time. **(B)** RMSE against hit rates, which are the average number of odor hits per second.

Figure 7B shows the cumulative density function of the actual rewards Δ*S* for matching and mismatching behaviors. As shown in the figure, mismatching behaviors have a higher probability of greater entropy reductions (particularly values of approximately 10^−4^ and 10^−1^). Mismatching behaviors also have a higher probability of a larger decrease in entropy (values of approximately -4 × 10^−3^) as shown in Figure 7D. Figures 8A,B show the cumulative odor hits and hit rate against the root mean squared error between the actual Δ*S* and the expected reward *E*[Δ*S*]. This was calculated as shown in Equation (3), where *N* is 20 because the sampling frequency of the behavioral measurement system is 20 Hz.

(3) $$\begin{array}{l} RMSE=\sqrt{\frac{1}{N}\overset{N}{\underset{i\;=1}{\sum }}{\left(\Delta S_{i+1}-E\left[\Delta S_{i}\right]\right)}^{2}} \end{array}$$

## 5. Discussion

In this study, we investigated the possible causes of variability in the programmed behavior model of the male silk moth. Specifically, we asked whether such variability leads to higher information gains; in other words, if it minimizes the information entropy of the probability distribution of the moth regarding the location of an odor source. We also investigated whether the probabilistic framework of infotaxis can explain how the male silk moth selects maneuvers to balance the exploration and exploitation of the expected rewards.

### 5.1. Relationship Between Behavioral Variability and Information Rewards

In a recent study, Shigaki et al. (2019b) simultaneously measured the odor search behavior of male silkmoths and the neural activity from their lateral accessory lobe (LAL). The LAL generates motor commands in response to odor stimuli. That study found that silkmoths are less likely to “surge” (move forward) as the frequency of odor hits increases. In terms of infotaxis, this can be interpreted as moths preferring rotations (exploration) because, at high odor encounter rates, the expected decrease in entropy is less than at low rates. Our results found that matching and mismatching behaviors generate rewards at high and low hit rates, respectively (Figure 6). Thus, this leads us to believe that at high hit rates, silk moths prefer reactive or more exploitative behaviors, and at low rates, they prefer more stochastic or explorative behaviors such as rotations instead of straight forward moves. Furthermore, this tendency was observed on all specimens that reached the odor source.

An interesting interpretation of these results can also be made from the viewpoint of reinforcement learning (RL). In this field, an agent learns to behave according to an optimal policy with the highest expected accumulated reward over a time horizon. Nonetheless, many RL algorithms face the exploration and exploitation dilemma in which greedily selecting the actions with the highest reward can lead to suboptimal policies stuck in the local maxima. A common way to avoid this is to add stochasticity in the selection of actions; thus balancing exploration and exploitation, using methods such as ϵ-greedy algorithms (Sutton and Barto, 2018). An analogy can be made to the behavior of the silkmoth in the sense that some randomness in the selection of the “surge” maneuver leads to higher information gains and possibly a better odor source search performance. This can be clearly seen in Figures 7B,D, where the probability of obtaining better rewards is higher for the mismatching behaviors.

### 5.2. Exploration and Exploitation in Silk Moth Behavior

We found that maneuvers that deviate from the programmed behavior model correspond to a larger expected decrease in entropy, that is, a higher expected reward in the terminology of reinforcement learning. Therefore, we demonstrated the capability of the infotaxis strategy to quantitatively express maneuvers that deviate from the programmed behavior as explorative and those that match it as exploitative.

Another interesting point to note is the relationship between matching and mismatching behaviors with the root mean squared error (RMSE) of the real vs. expected rewards. As shown in Figure 8A, the error decreases proportionally to the accumulation of odor hits. This is relatively intuitive because more detections narrow down the belief of the source location. However, more RMSE occurs between real and expected rewards at times of high hit rates. Furthermore, the matching behaviors have a lower error than the mismatching behaviors. One possible interpretation for this is that matching behaviors are more exploitative; thus they are more similar to the greedy infotaxis policy, whereas the mismatching behaviors are more explorative; hence, they differ from the expected reward of the infotaxis strategy.

We believe that being able to represent animal olfactory behavior through a method such as infotaxis is an important contribution to the fields of ethology and robotics because having a representation of the decision process of animals in terms of probabilistic beliefs and expected rewards facilitates the algorithmic implementation of these processes in robots. Furthermore, it allows for the refinement of these decision processes using tools such as machine and reinforcement learning.

## 6. Conclusion

In this study, we measured the behavior of moths using a virtual reality system that presents accurate and reproducible odor stimuli by using blue light and optogenetic moths. We then took trajectories from these measurements and modeled them as an infotaxis (Vergassola et al., 2007) strategy. We used infotaxis-based modeling to determine if variability in the silkmoth behavior is related to higher gains in information regarding the probabilistic distribution of the source location. We found that variations have a higher probability of obtaining larger information gains than “programmed behaviors” (i.e., reactive, exploitative behaviors). This suggests that silkmoths incorporate some stochasticity into their behavior to balance the exploration and exploitation of information gains. Future studies should be conducted to develop ways to extract decision-making mechanisms from free-running silkmoths. In this study, we used tethered moths walking on a treadmill, and, although such a device imposes minimal disturbances on the moth behavior, we believe it is necessary to study whether models from free-running experiments will differ from those in this specific study. It would also be useful to develop an olfactory search algorithm based on the silkmoth exploration/exploitation mechanisms elucidated in this paper and then implement such an algorithm on a robot to test whether the search performance is improved compared with either the programmed behavior or the infotaxis strategy.

## Data Availability Statement

The original contributions presented in the study are included in the article/Supplementary Files, further inquiries can be directed to the corresponding author/s.

## Author Contributions

CH-R, SF, SS, and DK contributed conception and design of the study. SF conducted the virtual reality silk moth experiments. CH-R performed the numerical analyses and wrote the manuscript. TS, RK, and HS provided genetically modified silk moths. All authors read and approved the submitted version.

## Conflict of Interest

SF was employed by MHPS Ltd. The remaining authors declare that the research was conducted in the absence of any commercial or financial relationships that could be construed as a potential conflict of interest.

## Appendix A: Infotaxis strategy

Herein, we provide a more detailed explanation of the derivation of the infotaxis formulae. We based this explanation on the work of Pang (2018) and the original infotaxis strategy developed by Vergassola et al. (2007). The agent's belief in the source location *P*(**r**_*src*_|$T_{t}$) can be written using Bayes' theorem, as indicated in Equation (A1).

(A1) $$\begin{array}{l} P\left({\text{r}}_{src}|T_{t}\right)=\frac{P\left(T_{t}|{\text{r}}_{src}\right)P\left({\text{r}}_{src}\right)}{P\left(T_{t}\right)}\propto P\left(T_{t}|{\text{r}}_{src}\right)P\left({\text{r}}_{src}\right) \end{array}$$

where *P*($T_{t}$|**r**_*src*_) is the likelihood of the source position and *P*(**r**_*src*_) is the prior distribution of the source. Infotaxis assumes that odor hits and misses are independent of one another and the likelihood of the source position takes the following form:

(A2) $$\begin{array}{l} P\left(T_{t}|{\text{r}}_{src}\right)=\underset{t}{\prod }P\left(h\left({\text{r}}_{t}\right)|{\text{r}}_{src}\right) \end{array}$$

where *h*(**r**_*t*_) is 1 if the agent detects an odor hit at time *t* and 0 if it detects a miss. The infotaxis strategy considers that the number of hits follows a Poisson distribution; hence, the probability of a hit or miss becomes the following:

(A3) $$\begin{array}{l} P\left(h\left({\text{r}}_{t}\right)=0|{\text{r}}_{src}\right)=\exp\left[-R\left({\text{r}}_{t}|{\text{r}}_{src}\right)\Delta t\right] \end{array}$$

(A4) $$\begin{array}{l} P\left(h\left({\text{r}}_{t}\right)>0|{\text{r}}_{src}\right)=1-P\left(h\left({\text{r}}_{t}\right)=0|{\text{r}}_{src}\right) \end{array}$$

where *R*(**r**_*t*_|**r**_*src*_)Δ*t* is the mean rate of hits the agent expects at **r**_*t*_, during a time period of Δ*t*, given a source position **r**_*src*_. The Supplementary Material of the original paper on infotaxis indicate that the hit rate is derived from the advection-diffusion equation of a turbulent plume and define it as follows:

(A5) $$\begin{array}{l} R\left(\text{r}|{\text{r}}_{src}\right)=\frac{E}{\ln\;\left(\frac{\lambda }{\alpha }\right)}e^{\frac{\left(x_{src}-x\right)V}{2D}}K_{0}\left(\frac{\left|\text{r}-{\text{r}}_{src}\right|}{\lambda }\right) \end{array}$$

(A6) $$\begin{array}{l} \lambda =\sqrt{\frac{D\tau }{1+V^{2}\tau /\left(4D\right)}} \end{array}$$

where *E* is the emission rate of odor particles, which have an effective diffusivity *D* and, a finite lifetime τ, and are advected by a wind with mean velocity *V* that blows in the positive *x*-direction. *K*_0_ is the modified Bessel function of order zero, and α is the radius of a round-shaped agent. In our calculations of the information entropy of the silkmoth we used the following parameters into Equation A6: α=10 mm, *E*=1, τ=6.3 s, *D*=0.012, and *V*=0.1 m/s to match the wind speed in the moth experiments. The range of possible values for the source location was a 1730 × 770 lattice; i.e. the size of each cell was 0.26 mm. For Equation A4 we set Δ*t* to 50 ms; which is the same as the sampling period of the treadmill described in section 3.1. At each time step, the belief of the source position distribution *P*($T_{t}$|**r**_*src*_) can be recursively updated as follows:

(A7) $$\begin{array}{l} P\left({\text{r}}_{src}|T_{t+1}\right)=P\left(h\left({\text{r}}_{t+1}\right)|{\text{r}}_{src}\right)P\left({\text{r}}_{src}|T_{t}\right) \end{array}$$

At each time step, the agent considers five possible actions: moving forward, backward, left, right, or waiting. For each possible action, it calculates the probability *p*_*_ that the action will result in finding the source:

(A8) $$p^{*}=\sum_{{\text{r}}_{src}}P\left({\text{r}}_{src}|T_{t}\right)P\left(\left|{\text{r}}^{\prime }-{\text{r}}_{src}\right|\approx 0\right)$$

Consequently, the probability of not finding the source is 1 − *p*_*_. If the source is found, then the entropy of the belief will become zero, that is, Δ*S*^*^=(0 − *S*_*t*_)=−*S*_*t*_. To balance the exploration and exploitation, the agent also considers the case in which it does not find the source after taking an action. In such case, it would sample from the environment either a miss with a probability *p*_*m*_ or a hit with a probability *p*_*h*_=1 − *p*_*m*_. The probability of sampling a miss is the average of the miss probability over the range of possible source locations:

(A9) $$\begin{array}{l} p_{m}=\underset{{\text{r}}_{src}}{\sum }P_{m}\left({\text{r}}_{src}|T_{t}\right)=\underset{{\text{r}}_{src}}{\sum }P\left(h\left({\text{r}}^{\prime }\right)=0|{\text{r}}_{src}\right)P\left({\text{r}}_{src}|T_{t}\right) \end{array}$$

where **r**′ is the future position of the agent after taking an action. The agent also estimates how its source position belief, as well as its entropy, would change after moving. The change in entropy after sampling a miss or a hit at **r**′ would be the following:

(A10) $$\begin{array}{l} \Delta S_{m}=-\underset{{\text{r}}_{src}}{\sum }P_{m}\left({\text{r}}_{src}|T_{t}\right)\ln\left(P_{m}\left({\text{r}}_{src}|T_{t}\right)\right)-S_{t} \end{array}$$

(A11) $$\begin{array}{l} \Delta S_{h}=-\underset{{\text{r}}_{src}}{\sum }P_{h}\left({\text{r}}_{src}|T_{t}\right)\ln\left(P_{h}\left({\text{r}}_{src}|T_{t}\right)\right)-S_{t} \end{array}$$

Overall, the agent calculates the expected change of entropy by moving from **r**_*t*_ to **r**′ as follows:

(A12) $$\begin{array}{l} E\left[\Delta S\left({\text{r}}_{t}\mapsto {\text{r}}^{\prime }\right)\right]=p^{*}\left(0-S_{t}\right)+\left(1-p^{*}\right)\left(p_{m}\Delta S_{m}+p_{h}\Delta S_{h}\right) \end{array}$$

where the terms on the left and right sides of the sum are the change in entropy if the source is found or not found at **r**′, respectively. Finally, the agent chooses the action *a* with the largest expected decrease in entropy (see Figure 3D) as:

(A13) $$\begin{array}{l} a={\arg\min}_{{\text{r}}^{\prime }}\left(E\left[\Delta S\left({\text{r}}_{t}\mapsto {\text{r}}^{\prime }\right)\right]\right) \end{array}$$

After making a move, the agent encounters either a miss or a hit from the odor plume and updates the probability distribution of the source location. The agent then repeats the navigation policy process iteratively until it finds the source.
